# Adherence to hyperbilirubinemia guidelines by midwives, general practitioners, and pediatricians in Indonesia

**DOI:** 10.1371/journal.pone.0196076

**Published:** 2018-04-19

**Authors:** Mahendra T. A. Sampurna, Kinanti A. Ratnasari, Risa Etika, Christian V. Hulzebos, Peter H. Dijk, Arend F. Bos, Pieter J. J. Sauer

**Affiliations:** 1 Department of Pediatrics, Dr. Soetomo General Hospital, Faculty of Medicine, Airlangga University, Surabaya, Indonesia; 2 Department of Pediatrics, Beatrix Children Hospital, University Medical Center Groningen, University of Groningen, Groningen, the Netherlands; TNO, NETHERLANDS

## Abstract

Severe hyperbilirubinemia, which may result in kernicterus, is seen more frequently in low and middle-income countries, such as Indonesia, than in high-income countries. In Indonesia midwives, general practitioners (GPs), and pediatricians are involved in the care of jaundiced newborn infants. It is unknown whether the high incidence of severe hyperbilirubinemia in this country is related to a lack of awareness of existing hyperbilirubinemia guidelines issued by, for example, the World Health Organization, the American Academy of Pediatrics, or the Indonesian Health Ministry, or to a lack of adherence to such guidelines. The aim of this questionnaire study was to assess health professionals’ awareness of existing guidelines and their adherence to these guidelines in daily practice. We handed out a ten-question questionnaire to midwives, GPs, and pediatricians that included questions about the professionals themselves as well as clinical questions. The midwives completed 291 questionnaires, the GPs 206, and the pediatricians 154, all of which we used for our analysis. Almost 30% of the midwives and 23% of the GPs were either unaware of any existing guidelines or they did not adhere to them. Only 54% of the midwives recognized the warning signs of severe hyperbilirubinemia correctly, compared to 68% of the GPs and 89% of the pediatricians. Twenty-eight percent of the midwives and 31% of the GPs indicated that their first follow-up visit was after 72 hours, while 90% of them discharged infants after less than 48 hours after birth. The awareness of and adherence to guidelines for preventing and treating hyperbilirubinemia is low amongst the midwives and GPs in Indonesia. This may be an important contributing factor in the high incidence of severe hyperbilirubinemia in Indonesia.

## Introduction

Increased bilirubin levels are commonly seen in newborn infants and it is one of the leading causes for hospitalization during the first week after birth [1–7]. Early diagnosis of hyperbilirubinemia and phototherapy treatment can prevent brain damage, while late diagnosis and inappropriate or ineffective treatment may account for the disproportionately high incidence of bilirubin-induced acute encephalopathy and long-term morbidity seen in low and middle-income countries [4]. Worldwide, an estimated 481,000 term infants suffer from severe hyperbilirubinemia annually [5]. At least 75% of these infants live in South East Asia, China, and sub-Saharan Africa [5,6]. A recent survey in Indonesia indicated that the incidence of severe hyperbilirubinemia, defined as a total serum bilirubin of more than 340 μmol/L (20 mg/dL), is 7%. Acute bilirubin encephalopathy was found in 2% of the infants [7].

In Indonesia, the care of term-born infants during the first week after birth is provided primarily by midwives and general practitioners (GPs). The majority of infants are not born in a hospital, but in an obstetric clinic or at home. Several guidelines for diagnosing and treating hyperbilirubinemia are available in Indonesia. The Indonesian Ministry of Health (IHM) issued a guideline for midwives [8]. GPs often use the guideline issued by the World Health Organization (WHO) [9]. The Indonesian Pediatric Society advises its members to adhere to the guideline issued by the American Academy of Pediatrics (AAP) [10]. A part of the AAP guideline was translated into Bahasa Indonesia, the official language of Indonesia, and issued by the Indonesian Pediatric Collegium, the Board responsible for the education of pediatricians in Indonesia [10]. In addition to issuing the guidelines, phototherapy for hyperbilirubinemia was made available in almost the whole of Indonesia. Nevertheless, cases of severe hyperbilirubinemia are still frequently seen. We wondered whether this may be related to health professionals being unaware of the existence of these guidelines or to their failure to adhere to the recommendations contained in them.

With the help of a questionnaire, we investigated awareness of the guidelines in three groups of health professionals involved in the care of newborn infants: midwives, GPs, and pediatricians. We also assessed whether these health professionals adhered to the guidelines in daily practice.

## Methods

The study was approved by the Ethical Committee in Health Research of the Dr. Soetomo General Hospital Surabaya (number 390/ Panke.KKE/V/2017). The committee waived the need to ask consent, because data were obtained anonymously by questionnaires. In 2016 we handed out a questionnaire to midwives, GPs, and pediatricians at the end of a postgraduate teaching symposium, not related to hyperbilirubinemia, held by the Indonesian Pediatric Society (PIT IKA VIII Makassar) on East Java, Indonesia. We performed a pilot study by first handing out the questionnaires to 25 midwives, 25 GPs, and 25 pediatricians to check the clarity of the questions, variability, and reliability. The questionnaires for the three groups were identical as far the questions on hyperbilirubinemia were concerned. Only the question relating to the professionals’ place of practice was different, as midwifes do not work in NICUs. The questionnaire consisted of ten questions. First, we collected demographic background information on the respondents regarding age, number of years of practice, and the number of infants seen per month. This was followed by questions about the professionals’ awareness of guidelines on diagnosing and treating hyperbilirubinemia and if so, whether they adhered to the guidelines in practice. For each question the respondents could choose one of the three to five optional answers provided. Only one answer was allowed except in the case of Question 4 (S1 Table). Finally, a clinical case was presented: a 30-year-old mother, with blood group O+, gave birth to a 2.8 kg male infant with cephalic hematoma after 37 weeks of gestation. Before discharge, at 36 hours after birth, the infant appeared jaundiced. I would … (choose the one answer that best fits your usual practice) (S1 Table).

### Statistics

We present all the data as numbers and percentages. Using IBM SPSS Statistics, Version 21 (Chicago, IL, USA), we tested the differences for all the variables between the groups of health professionals for significance with Pearson’s chi-square tests. Probability values of *P <* 0.05 were considered statistically significant.

## Results

The questionnaires were handed out to 384 midwives, 250 GPs, and 593 pediatricians. The response rates were 303 (79%), 220 (88%), and 178 (30%), resepectively. Of these questionnaires a total of 651 (53%) were filled in completely: 291 by the midwives, 206 by the GPs, and 154 by the pediatricians. We used these questionnaires for further analysis.

Table 1 provides the demographic characteristics of the respondents. The midwives were either employed by obstetric clinics or private practices and they attended deliveries at home. GPs saw newborn infants at their practices, while pediatricians worked in obstetric clinics, hospitals, or in private practices. The midwives and GPs tended to be younger, had fewer years’ experience, and saw significantly fewer infants per month compared to the pediatricians (*P* < 0.05 in all three cases).

**Table 1:** Demographic characteristics of the respondents.

		Midwives	GPs	Pediatricians	*P* value*
		N = 291	N = 206	N = 154	
Age of the respondent (y)	≤ 30	169 (58)	116 (56)	0 (0)	0.01
	31–39	65 (22)	69 (34)	56 (36)	
	40–49	48 (17)	17 (7)	53 (35)	
	50–59	8 (3)	2 (1)	33 (21)	
	≥ 60	1 (0)	5 (2)	12 (8)	
Years of practice	≤ 1	39 (13)	25 (12)	9 (6)	0.01
	2–5	117 (40)	113 (55)	52 (34)	
	6–9	60 (21)	46 (22)	39 (25)	
	≥ 10	75 (26)	22 (11)	54 (35)	
Level of practice	Nursery	113 (39)	14 (7)	77 (50)	0.01
	NICU Level II	0 (0)	10 (5)	47 (30)	
	NICU Level III	0 (0)	8 (4)	12 (8)	
	Private Practice	178 (61)	174 (84)	18 (12)	
Number of infants treated every month	≤ 1	19 (6)	93 (45)	0 (0)	0.01
	2–5	133 (46)	71 (35)	10 (6)	
	6–9	46 (16)	25 (12)	12 (8)	
	≥ 10	93 (32)	17 (8)	132 (86)	

Data are presented as numbers and (percentages).

* *P* values are the results of Pearson’s chi-square tests between the groups of respondents for the categories of the demographic variables.

The AAP guideline was adhered to by 84% of the pediatricians, the WHO guideline by 46% of the GPs, and 54% of the midwives adhered to the IHM guideline (Table 2). Twenty nine percent of the midwives and 23% of the GPs were unaware of a hyperbilirubinemia guideline or they failed to adhere to one. Almost 50% of the pediatricians indicated that they had difficulty gaining access to the guidelines. Only 54% of the midwives named warning signs for severe hyperbilirubinemia correctly (i.e. jaundice less than 24 hours after birth and jaundice to the palms of the hands and the soles of the feet) compared to 68% of the GPs and 89% of the pediatricians (*P* < 0.05).

**Table 2:** Results of the survey.

		Midwives	GPs	Pediatricians	* *P* value
		N = 291	N = 206	N = 154	
Early recognition of jaundice	To palms and soles Before 24 h of age	44 (15) 112 (39)	102 (49) 39 (19)	77 (50) 60 (39)	0.01
	Pale-colored feces Between 24 h to 14 d Bilirubin levels of > 10 mg/dL	52(18) 82 (28) 1 (0)	24 (12) 22 (11) 19 (9)	6 (4) 3 (2) 8 (5)	
Predischarge bilirubin measurement	Yes, mostly	48 (17)	53 (26)	12 (8)	0.01
	Yes, if jaundice present	147 (50)	112 (54)	100 (65)	
	No	96 (33)	41 (20)	42 (27)	
Specific guideline used	IHM guideline	156 (54)	55 (27)	10 (7)	0.01
	WHO guideline	41 (14)	94 (46)	7 (4)	
	AAP guideline	3 (1)	7 (3)	129 (84)	
	NICE guideline	0 (0)	2 (1)	3 (2)	
	No guideline	84 (29)	48 (23)	0	
	Other	7 (2)	0 (0)	5 (3)	
Guideline access	Yes	205 (71)	142 (69)	80 (52)	0.01
	Not easy	85 (29)	64 (31)	74 (48)	
Problems in management	Education	112 (38)	22 (11)	49 (32)	0.01
	Diagnostics	15 (5)	44 (21)	32 (21)	
	Therapy	113 (39)	97 (47)	38 (24)	
	Facilities	51 (18)	43 (21)	35 (23)	
Case scenario	Discharge and follow- up	18 (6)	19 (9)	7 (4)	0.01
	Lab. tests bilirubin + BG	128 (44)	117 (57)	83 (54)	
	Cancel discharge and start phototherapy	25 (9)	21 (10)	62 (40)	
	Refer to pediatrician	120 (41)	49 (24)	2 (1)	
Discharge (LOS)	≤ 24 h	163 (56)	85 (41)	11 (7)	0.01
	25–48 h	99 (34)	111 (54)	45 (29)	
	> 48 h	29 (10)	10 (5)	98 (64)	
First follow-up	**≤** 24 h	14 (5)	10 (5)	0 (0)	0.01
	25–48 h	107 (36)	49 (24)	9 (6)	
	49–72 h	87 (30)	83 (40)	66 (43)	
	>72 h	83 (29)	64 (31)	79 (51)	

Data are numbers and (percentages).

* *P* values are the results of Pearson’s chi-square tests between the three types of health professionals in all categories of the variables.

Abbreviations: IHM—Indonesian Health Ministry, WHO -World Health Organization, AAP -American Academy of Pediatrics, NICE—National Institute for Health and Care Excellence, BG—blood group, rhesus and Coombs testing, LOS—length of stay.

The majority of the midwives (90%) and GPs (95%) discharged the infants less than 48 hours after birth, while approximately two-thirds of the pediatricians (64%) discharged infants more than 48 hours after birth (*P* < 0.05). Twenty-nine percent of the midwives and 31% of the GPs indicated that the first follow-up visit was only after 72 hours, while infants were discharged rather soon after birth. Fifty-three percent of the respondents who had indicated that they discharged infants within 24 hours after birth scheduled the first follow-up visit after more than 48 hours, while 22% did so after more than 72 hours (Table 3).

**Table 3:** Timing of first follow-up visit related to discharge (length of stay).

First follow-up	Discharge (LOS)			*P* value
	≤ 24 h n (%)	25–48 h n (%)	> 48 h n (%)	
**≤** 24 h	17 (7)	7 (3)	0 (0)	0.01
25–48 h	105 (40)	43 (17)	17 (12)	0.01
49–72 h	81 (31)	106 (41)	49 (36)	0.01
>72 h	56 (22)	99 (39)	71 (52)	0.01

Data are numbers and (percentages).

*The *P* value is the result of the Pearson’s chi-square tests between the timing of discharge and the timing of the first follow-up visit.

Abbreviation: LOS—length of stay.

## Discussion

In this questionnaire study we found that 29% of the midwives and 23% of the GPs in our sample were unaware of the existence of any guidelines to diagnose and treat hyperbilirubinemia in newborn infants. Moreover, 46% of the midwives and 32% of the GPs did not recognize the warning signs of clinically relevant hyperbilirubinemia correctly. Finally, almost 60% of the midwives and more than 70% of the GPs scheduled a follow-up visit more than 48 hours after early discharge, while the guidelines recommended seeing the infants again at least within 48 hours. These factors may contribute to the higher incidence of severe hyperbilirubinemia found in Indonesia compared to high-income countries.

A number of studies found that severe hyperbilirubinemia is more frequent in low and middle-income countries [4,5,11]. This may be caused either by limited availability of phototherapy or to late diagnosis of hyperbilirubinemia. In Indonesia, phototherapy is available in most parts of the country, although it is uncertain whether the intensity of phototherapy is sufficient everywhere. Hyperbilirubinemia is usually diagnosed by clinical assessment of the infant, for instance, by using the Kramer jaundice score [12]. Maisels and colleagues reported that this score can help to identify infants with increased bilirubin levels, but it is not helpful in estimating the levels of bilirubin [13]. A bilirubin measurement, either transcutaneous or in blood, is necessary to identify the infants who need treatment. During the first days after birth, daily visits by health care workers are needed to detect the infants at risk of severe hyperbilirubinemia and whose bilirubin levels need to be checked.

Previous studies in high-income countries also found that guidelines to prevent, diagnose, and treat hyperbilirubinemia are not always adhered to [14–17]. Tartaglia and colleagues [15] used a compliance score to measure adherence to the AAP guideline in the Children’s Hospital in Columbus, Ohio, USA. The compliance score increased from 60% before to 90% after an intervention. The campaign aimed at increasing the awareness of the guidelines. Atkinson and colleagues [14] found that pediatricians only provided phototherapy to 54% of the infants who should have received it in accordance with the AAP guidelines. Darling and colleagues [16] investigated the implementation of new guidelines drawn up by the Canadian Pediatric Society in hundred Canadian hospitals. Seventy-nine hospitals indicated that they had implemented the guidelines. Nevertheless, despite it being recommended in the guideline, only 70% of the hospitals implemented measuring bilirubin levels before discharge. Sgro and colleagues [17] demonstrated that introducing guidelines does indeed help to reduce the incidence of severe hyperbilirubinemia. They found that after the Canadian guidelines were introduced the incidence of severe hyperbilirubinemia decreased from 1 in 2480 to 1 in 8352 live-births.

There are a number of reasons why guidelines may go unheeded or why they are not adhered to. Cabana and colleagues [18] identified the following barriers that prevent health care professionals from adhering to clinical practice guidelines: lack of awareness, lack of familiarity, lack of agreement, lack of self-efficacy, lack of outcome expectancy, lack of motivation to change current practice, and external barriers that affect the ability to put a recommendation into practice. Most of these barriers apply to the situation in Indonesia. We found that 29% of the midwives and 23% of the GPs were unaware of the guidelines. This is a serious problem because these health care workers take care of the majority of the millions of infants born in Indonesia every year. Lack of agreement, lack of self-efficacy, and lack of outcome expectancy may well play a role in the case of Indonesian midwives and GPs. Midwives advocate exclusive breastfeeding, without supplementing it with formula. At the same time, breastfeeding is an important risk factor for the development of hyperbilirubinemia, particularly if nursing is not going well. Midwives may not be fully aware of the risks of severe hyperbilirubinemia because they do not follow the infants for any length of time (only the first week) and have limited experience as expressed by the number of newborn infant cared for. Insufficient training may also play a role. The same risk factors may apply to GPs. One third of the pediatricians indicated not to measure the bilirubin level before discharge, even though it is recommended in the original AAP guideline and repeated in a recent review [3]. Such lack of adherence may be due either to disagreement with the recommendation or the lack of motivation to change current practice. It is unlikely to be related to external barriers. Pediatricians seem to rely on their clinical judgment, while it is known that clinical evaluation cannot reliably distinguish between bilirubin levels that need treatment versus levels that do not require treatment [3,10].

There are a number of well-known risk factors for developing severe hyperbilirubinemia in late preterm and term infants: a gestational age of less than 38 weeks, exclusive breastfeeding, jaundice during the first 24 hours, a previous sibling or siblings with jaundice, cephalohematoma or significant bruising, and hemolytic diseases [3,10,11]. Being born to an Asian mother is also a known risk factor. The lack of knowledge regarding risk factors that we found amongst a high percentage of midwives may also contribute to the high incidence of severe hyperbilirubinemia in Indonesia. These factors are of special interest when newborn infants are discharged within 48 hours after birth. A home visit by an experienced health professional within 24 hours is recommended in case of risk factors [19].

It is tempting to speculate that the existence of different guidelines might be part of the identified problems. The incidence of hyperbilirubinemia might be reduced if a single, uniform guideline were to be introduced that applies to the whole of Indonesia, and that is adhered to by all Indonesian midwives, GPs and pediatricians. An added benefit of a nation-wide guideline would be that whenever health care professionals move to a different part of the country, they nevertheless continue to use the same guideline instead of having to learn to use a different one. We plead for a guideline that addresses not only the pathophysiology of hyperbilirubinemia and its neurological sequelae, but also one that streamlines the way health care for newborns is organized in Indonesia. In our opinion, uncritically adopting the guidelines used in high-income countries is not appropriate, because Indonesian demographics are unique and access to its health care system is organized differently.

Our study has a number of limitations. First, inspired by the study of Mateo and colleagues [20] and because other validated questionnaires were not available, we developed and used our own, non-validated questionnaires. We did, however, first evaluate the questions and confirmed that they were clear and could be answered consistently. A second limitation is that our survey was limited to health professionals in one area of Indonesia, thus we cannot be sure that our results apply to Indonesia as a whole. The midwives, GPs, and pediatricians who responded to our questionnaires comprised a relatively small proportion of the neonatal health care workers in our area. We doubt, however, whether covering a larger area or a larger group of participants would have changed the outcome of our survey. Finally, we did not determine whether the health professionals in fact practiced what they had indicated in the questionnaires and we did not check medical records.

## Conclusion

Awareness of and adherence to guidelines for preventing and treating hyperbilirubinemia is relatively low in midwives and GPs in Indonesia and needs to be addressed. We assume that lack of awareness and lack of adherence play an important role in the high incidence of severe hyperbilirubinemia in Indonesia. We recommend introducing a single, nation-wide guideline to be used by all health care professionals in Indonesia who care for newborn infants in order to reduce the incidence of hyperbilirubinemia in Indonesia.

## Supporting information

S1 Table Questionnaire on neonatal hyperbilirubinemia management on East Java, Indonesia (Translated from Bahasa Indonesia to English). (DOCX)
