# Supplementary material for: Adherence to hyperbilirubinemia guidelines by midwives, general practitioners, and pediatricians in Indonesia
Source: PLoS One. 2018 Apr 19;13(4):e0196076. doi: 10.1371/journal.pone.0196076 (PMC5909511; doi:10.1371/journal.pone.0196076)
Supplement: S1 Table — (DOCX) [file pone.0196076.s001.docx]

**Supporting information**

**Table 1.** **Questionnaire on** the management of n**eonatal** h**yperbilirubinemia on** **East** **Java,**

**Indonesia** (Translated from Bahasa Indonesia to English)

| **No.** | **Question** |  | **Answer** |  |
| --- | --- | --- | --- | --- |
|  |  | Please encircle the one answer that best describes your practice | |  |
|  |  |  |  |  |
|  |  |  | |  |
| 1 | What is your profession? | A. Midwife | |  |
|  |  | B. General practitioner | |  |
|  |  | C. Pediatrician | |  |
|  | How old are you? | A. ≤ 30 | |  |
|  |  | B. | 31 - 39 |  |
|  |  | C. | 40 - 49 |  |
|  |  | D. | 50 - 59 |  |
|  |  | E. | ≥ 60 |  |
|  | How many years have you been working | A. ≤ 1 | |  |
|  | in your profession? | B. | 2 - 5 |  |
|  |  | C. | 6 - 9 |  |
|  |  | D. ≥ 10 | |  |
|  | Where do you work the most (highest | A. Nursery | |  |
|  | level of NICU or practice)? | B. NICU Level II | |  |
|  |  | C. NICU Level III | |  |
|  |  | D. Private practice | |  |
| 2 | On average, how many newborns do you care for each month? | A. | ≤ 1 |  |
|  |  | B. | 2 - 5 |  |
|  |  |  |  |  |
|  |  | C. | 6 - 9 |  |
|  |  | D. ≥ 10 | |  |
| 3 | On average, at what age do you discharge a healthy, term and vaginally-born newborn | A. ≤ 24 | |  |
|  | (state the length of stay in hours)? | B. | 25 - 48 |  |
|  |  | C. > 48 | |  |
|  |  |  | |  |
| 4 | What do you consider warning signs of severe hyperbilirubinemia? | A. Visual: the palms of the hand and the soles of the feet are yellow | |  |
|  |  |  |  |  |
|  |  | B. Jaundice within < 24 hours after birth | |  |
|  |  | C. Jaundiced baby with pale stools, fever | |  |
|  |  | D. Jaundice between 24 hours and 14 days of age, baby active and growing | |  |
|  |  |  |  |  |
|  |  | E. Bilirubin level > 10 mg/dL in term infant | |  |
| 5 | Do you measure a baby’s bilirubin level before discharge? | A. Yes, as a rule | |  |
|  |  | B. Yes, if the baby looks jaundiced | |  |
|  |  | C. No, I never do | |  |

| 6 | At what age in hours do you schedule the first post-discharge follow-up (state the number of hours)? | A. | ≤ 24 | |
| --- | --- | --- | --- | --- |
|  |  | B. | 25 - 48 | |
|  |  | C. | 49 - 72 | |
|  |  | D. > 72 | | |
| 7 | Do you use a specific guideline regarding | A. Yes: Please encircle the guideline you | | |
|  | the management of hyperbilirubinemia? |  | usually use: | |
|  |  |  | 1) Basic Neonatal Essential Care (Indonesian | |
|  |  |  | Ministry of Health, IHM) | |
|  |  |  | 2) Hospital Care for Children (World Health Organization, WHO) | |
|  |  |  | 3) The guideline issued by the American Academy of Pediatrics (AAP) | |
|  |  |  | 4) The guideline issued by the National Institute for Health and Clinical Excellence (NICE) | |
|  |  |  |  | |
|  |  |  | 5) Other(s). State the name(s) …. | |
|  |  | B. | No |  |
| 8 | Do you have easy access to that guideline? | A. Yes, by means of: | | |
|  |  |  | 1) | Book |
|  |  |  | 2) | Online |
|  |  |  | 3) | Notes |
|  |  | B. | No | |
| 9 | What kind of problems do you encounter | A. Education and communication | | |
|  | most with regards to the management of | B. Diagnostics | | |
|  | hyperbilirubinemia? | C. Therapy | | |
|  |  | D. Facilities | | |
| 10 | A 30-year-old mother, blood group O^+^, | A. Discharge the baby and schedule a follow-up | | |
|  | gave birth to a 2.8 kg male infant with cephalohematoma after 37 weeks of |  | as an outpatient; | |
|  | gestation. Before discharge, at 36 hours | B. Ask for lab testing: blood typing, rhesus, | | |
|  | of age, the infant appeared jaundiced. I |  | coombs and bilirubin level measurement | |
|  | would (choose one answer that best fits your practice): | C. Cancel discharge and start phototherapy | | |
|  |  | D. Refer the baby to a pediatrician | | |
|  |  |  |  |  |
|  |  |  |  |  |
